# 

**Published:** 1899-06-24

**Authors:** 


					THE HOSPITAL. !? The standard of highest purity."?Lancet, June 24th, 1899- \J
CADBURY'S rf* M ** OADBURY'S
<-\ , COCOA IL ^ COCOA
ABSOLUTELY PURE. ^ NO DRUGS OR ALKALIES USED.
URW1CH UBSWHAL
iNFmMARr
No. <<65./ Vol. XXVI.
Saturday, June 24th, 1899.
[SubsCBrceP^?203.ermS'] PBICE TWOPENCE.

				

